# FABIAN-variant 2026: improved prediction of the effects of DNA variants on transcription factor binding

**DOI:** 10.1093/nar/gkag449

**Published:** 2026-07-07

**Authors:** Robin Steinhaus, Peter N Robinson, Dominik Seelow

**Affiliations:** Berlin Institute of Health at Charité – Universitätsmedizin Berlin, 10117 Berlin, Germany; Institute of Medical Genetics and Human Genetics, Charité – Universitätsmedizin Berlin, corporate member of Freie Universität Berlin and Humboldt-Universität zu Berlin, 13353 Berlin, Germany; Berlin Institute of Health at Charité – Universitätsmedizin Berlin, 10117 Berlin, Germany; The Jackson Laboratory for Genomic Medicine, Farmington, CT 06032, United States; Berlin Institute of Health at Charité – Universitätsmedizin Berlin, 10117 Berlin, Germany; Institute of Medical Genetics and Human Genetics, Charité – Universitätsmedizin Berlin, corporate member of Freie Universität Berlin and Humboldt-Universität zu Berlin, 13353 Berlin, Germany

## Abstract

Variants in promoters and enhancers can alter the binding of transcription factors (TFs), but their functional assessment remains difficult. FABIAN-variant is a web application that predicts the effects of DNA variants on TF binding by comparing position weight matrix (PWM) and transcription factor flexible model (TFFM) scores between reference and variant alleles. Here, we present FABIAN-variant 2026, a major update that expands the prediction model library from ~5000 to over 40 000 models for >1500 human TFs, sourced from nine PWM databases and including 1290 TFFMs. The application now supports the mouse genome (GRCm38 and GRCm39) with over 35 000 models for >1100 mouse TFs. An optional BPNet deep learning scorer provides neural network-based binding predictions for 240 human TFs. Known TF binding site information has been expanded from three to five sources. Predictions for over 1400 heterodimer TF complexes have been added. The web server has been rewritten in Rust and the scoring engine optimized, reducing runtime by ~70%. A RESTful JSON API and a standalone command-line version enable programmatic access and local high-throughput analysis. FABIAN-variant 2026 is available at https://fabianapp.org/variant26/. The web server is free and open to all users and there is no login requirement.

## Graphical Abstract

**Graphical Abstract:**
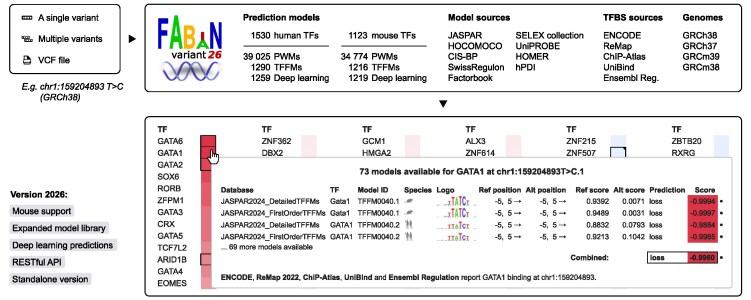

## Introduction

While many tools exist for assessing the disease-causing potential of coding variants, coding regions make up only ~1.5% of the human genome. Predicting the effect of variants in noncoding regions is much less straightforward, but such variants can also cause disease, e.g. by impairing the binding of transcription factors (TFs) to promoters or enhancers, thereby leading to changes in gene expression. In 2022, we published FABIAN-variant (FAst BInding-site ANalysis) to provide a simple way for researchers and clinicians to predict the potential effect of noncoding variants (single nucleotide variants and short indels) in promoters and enhancers on TF binding [1]. For each variant, the application evaluates position weight matrices (PWMs) and transcription factor flexible models (TFFMs) in a sliding window around the variant position, scoring the reference and the variant sequence on both strands. For each model, the resulting scores are combined into a single score between $-1$ (predicted loss of binding) and $+1$ (predicted gain). When multiple models are available for a TF, the individual scores are averaged into a combined score. The application supports single variants, variant lists, and VCF files, and allows filtering by known TF binding sites (TFBSs).

Since its publication, FABIAN-variant has been applied in functional genomics and clinical genetics. Its predictions of altered TF binding have been experimentally confirmed, including reduced HNF1A binding at *HNF4A* promoter variants causing MODY diabetes [2] and decreased GATA3 binding at a neuroblastoma risk locus [3]. The application has also been incorporated into a variant prioritization pipeline for undiagnosed rare disease [4]. MutationTaster2025 [5] includes a link to FABIAN-variant for each analysed variant.

Here we describe FABIAN-variant 2026, a major update. The key improvements are: (i) an approximately eight-fold expansion of the prediction model library; (ii) support for the mouse genome; (iii) deep learning-based binding predictions using BPNet; (iv) known TFBS information from five sources; (v) predictions for over 1400 heterodimer TF complexes; (vi) a rewritten backend with reduced runtime; (vii) a RESTful API; and (viii) a standalone version for local execution.

## Expanded prediction model library

The prediction model library has been expanded from ~5000 models for 1387 human TFs to 40 315 models for 1530 human TFs and 35 990 models for 1123 mouse TFs (Table 1). The previous version obtained most PWMs from MotifDb [6], an aggregated collection. All models are now retrieved directly from their original sources: nine PWM databases (39 025 human PWMs) and the JASPAR 2024 TFFM collection (1290 TFFMs).

**Table 1.** Prediction model sources in FABIAN-variant 2026. Models are assigned to each organism by matching TF gene symbols against reference lists from HOCOMOCO and CIS-BP. Models matching both species are included in both sets

Source	Type	Models for	Models for
		human TFs	mouse TFs
JASPAR 2026$^{1}$	PWM	3357	2903
HOCOMOCO v14	PWM	6307	5044
CIS-BP 3.00	PWM	6473	5580
SwissRegulon	PWM	1316	1313
Factorbook	PWM	15 061	13 682
SELEX collection$^{1}$	PWM	4635	4414
UniPROBE$^{1}$	PWM	1403	1403
HOMER$^{1}$	PWM	333	318
hPDI	PWM	140	117
JASPAR 2024$^{1}$	TFFM	1290	1216
**Total**		**40 315**	**35 990**

$^{1}$ Includes heterodimer TF complexes. Other sources contain only single-TF models. See the “Heterodimer TF complexes” section for per-source counts.

Several databases have been updated to their latest versions: CIS-BP [7] from 1.02 to 3.00, HOCOMOCO [8] from v11 to v14, and JASPAR [9] from 2022 to 2026. The SELEX collection has been expanded from [10] alone to five *in vitro* binding studies: [10, 11, 12, 13, 14]. HOMER [15] is a new addition. SwissRegulon [16], UniPROBE [17], and hPDI [18] have been retained. The largest single source is Factorbook [19], which contributes 15 061 PWMs derived from ENCODE ChIP-seq and HT-SELEX experiments.

TFFMs [20] are based on hidden Markov models and, unlike PWMs, can capture dependencies between adjacent positions. The 1290 TFFMs comprise 645 detailed and 645 first-order models from JASPAR 2024 [21], the most recent JASPAR release to include TFFMs. FABIAN-variant also incorporates 346 “motif patterns” (238 primary and 108 alternative) from the JASPAR 2026 deep learning collection, derived from BPNet [22] models via TF-MoDISco [23]. These are scored using the same log-likelihood framework as all other PWMs.

## Mouse genome support

The 2022 version of FABIAN-variant supported only the human genome (GRCh37 and GRCh38). The 2026 version adds the murine genome in versions GRCm38 (mm10) and GRCm39 (mm39).

A total of 35 990 prediction models are available for 1123 mouse TFs, extracted from the same databases as those for human TFs (Table 1). An optional same-species filter restricts predictions to models derived from data for the selected organism. Because most databases primarily contain human data, this filter substantially reduces coverage for the mouse. A human/mouse organism selector on the search page dynamically updates the available TFs, model counts, and genome assemblies. VCF files with mouse variants can be filtered by strain frequency data from the Mouse Genomes Project [24], analogous to gnomAD [25] filtering for human variants.

The positions of known TFBSs for the mouse genome were downloaded from ENCODE, ReMap, ChIP-Atlas, and UniBind for mm10, and from ReMap and Ensembl Regulation for mm39.

## Deep learning predictions

In addition to PWM and TFFM scoring, FABIAN-variant 2026 offers optional deep learning predictions using 1259 BPNet [22] convolutional neural network (CNN) models from the JASPAR 2026 collection [9], covering 240 human TFs. These models predict the ChIP-seq read coverage of a TF from sequence, both as a total read count and as a per-base binding profile. Because each model was trained on a specific ENCODE ChIP-seq dataset, predictions are cell-type-specific. As with the PWMs and TFFMs, the BPNet models are also mapped to mouse orthologs, yielding 1219 models for 209 mouse TFs.

**Figure 1.**
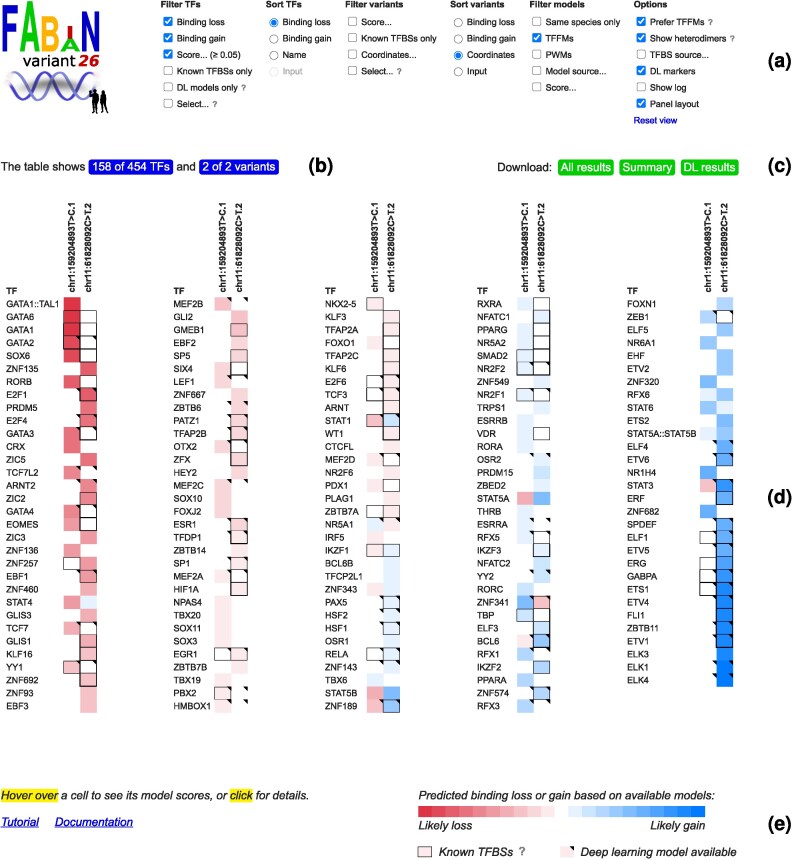
Results page for two promoter variants: chr1:159204893 T$> $C (rs2814778), reported to disrupt GATA1 binding at the *ACKR1* promoter [47], and chr11:61828092 C$> $T (rs968567), reported to facilitate ELK1 binding at the *FADS2* promoter [48]. (**a**) Filter, sort, and display options. (**b**) Currently displayed: 158 of 454 TFs and both variants. (**c**) Download options. (**d**) Heatmap of predictions, with variants as columns and TFs as rows, grouped into five panels. Shaded cells encode the combined score (red = loss, blue = gain). Deeper shades represent stronger predicted loss or gain. A cell border indicates a known TFBS at the variant position. A triangle marks TFs with BPNet deep learning predictions. (**e**) Legend.

**Figure 2.**
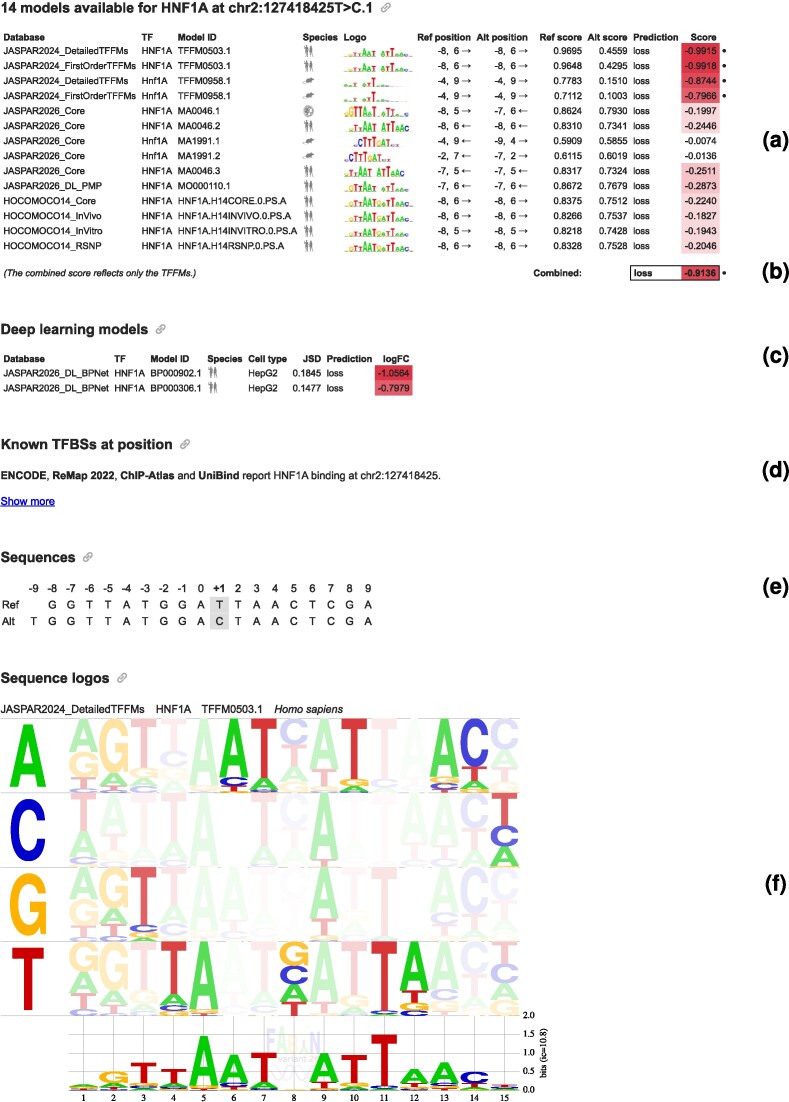
Detailed results page for HNF1A at the *PROC* promoter variant chr2:127418425 T>C (rs2104934553), reported to disrupt the hepatocyte nuclear factor 1 binding site in this liver-specific promoter [49]. The page is shown by clicking a cell in the results heatmap (Fig. 1d). (**a**) Per-model scores: four TFFMs and ten PWMs from JASPAR and HOCOMOCO. Each row gives the reference and variant scores, and the resulting score from −1 (loss) to +1 (gain). (**b**) Combined score, the average of the four TFFM scores. Contributing models are marked with • (TFFMs are preferred by default). (**c**) BPNet deep learning predictions: Jensen–Shannon distance and log2 fold change in predicted read counts. Both available models are trained on the liver cell line HepG2 and predict strong loss. (**d**) Known TFBS sources reporting HNF1A binding at this position: ENCODE, ReMap 2022, ChIP-Atlas, and UniBind. (**e**) Reference and variant sequences, with the variant at position +1. (**f**) Sequence logos, one per model (only the first shown).

For each variant–TF pair, FABIAN-variant reports two complementary BPNet-derived metrics per model, capturing the overall change in binding strength and the change in binding-profile shape (see the “Materials and methods” section). Both single nucleotide variants and indels are supported. On the results page, TFs with BPNet predictions are marked with a triangle (Fig. 1). The detailed results page for each variant–TF pair shows the metrics per cell line (Fig. 2c).

## Known TF binding site sources

Known TFBS data has been expanded to five sources: ENCODE [26], ReMap 2022 [27], ChIP-Atlas [28], UniBind [29], and Ensembl Regulation [30]. ENCODE, ReMap, and ChIP-Atlas provide experimentally determined TF binding regions from ChIP-seq data. UniBind identifies direct binding sites within ChIP-seq peaks, and Ensembl Regulation provides computationally predicted motif features within candidate regulatory regions. On the search page, users can choose which of these sources to include. On the results page, hovering over a known TFBS indicator shows which sources annotate the variant position, and a filter allows restriction to a specific source.

## Heterodimer TF complexes

FABIAN-variant 2026 includes prediction models for 1420 heterodimer TF complexes in human and 1355 in mouse, representing cooperative binding by TF pairs (e.g. AHR::ARNT, BACH1::MAFK, FOXA1::AR). Heterodimer PWMs are contributed by JASPAR 2026 (628 human/603 mouse PWMs), CAP-SELEX studies from [11, 14] (1959/1866), HOMER (3/3), and UniPROBE (2/2). JASPAR 2024 contributes 8 heterodimer TFFMs available for both organisms. Three display modes allow users to (i) merge heterodimer predictions with their component TFs, (ii) show heterodimers as separate entries, or (iii) exclude them entirely.

## Technical improvements

*Backend*. The web application backend has been rewritten from Perl CGI scripts with Slurm job scheduling to an asynchronous Rust HTTP server, eliminating per-request overhead and job queue latency. The PWM and TFFM scoring engine remains in C++ and has been optimized. Together, these changes reduce runtime by ~70% (Table 2). Progress updates are pushed via WebSocket connections.

**Table 2.** Runtime comparison between FABIAN-variant 2022 and 2026, measured in a headless Chromium browser (mean of three runs)

Workload	2022	2026	Reduction
Single variant	4.6 s	1.4 s	69%
Batch, 100 variants	1 min 48 s	30 s	72%
VCF, 10 000 variants	2 h 9 min	30 min 43 s	76%

Both versions used similar numbers of PWMs and TFFMs.

*RESTful API*. A RESTful API provides structured JSON responses for programmatic access, supporting variant submission, job status polling, retrieval of results, and job deletion. API documentation is available on the web server, where requests can be tested directly in the browser.

*Standalone version*. A standalone command-line version is available as a self-contained C++ binary for Linux, macOS, and Windows. Prediction models from openly licensed sources are embedded in the binary, enabling local execution for high-throughput analysis and for sensitive data that cannot be transmitted to an external server.

## Comparison with related tools

Several tools predict the effects of DNA variants on TF binding using PWMs: PERFECTOS-APE [31] evaluates variants through a web interface and standalone tool. motifbreakR [32] is an R/Bioconductor package for scoring variants against PWMs. RSAT variation-tools [33] provides variant scanning through the RSAT web platform and as command-line software. atSNP [34] uses importance sampling to assess the statistical significance of variant effects on TF binding. SNP2TFBS [35] is a pre-computed database of variants annotated with their predicted effects on TF binding. sTRAP [36, 37] uses a biophysical model to score variant effects on TF-binding affinity, and is available through a web interface and as software.

Other computational approaches train machine learning models on experimental chromatin data. DeepSEA [38] uses a deep CNN trained on chromatin-profiling data to predict variant effects on TF binding and other chromatin features, and is accessible through a web server and a public API. deltaSVM [39] applies support vector machines trained on accessible chromatin data to predict cell-type-specific variant effects, and is available as a command-line tool. ChromBPNet [40] uses base-resolution CNNs trained on ATAC-seq or DNase-seq data to predict cell-type-specific variant effects on chromatin accessibility, and is distributed as a Python package.

A separate class of tools applies large-scale sequence models to score variants. AlphaGenome [41] is a sequence-to-function model that jointly predicts thousands of functional genomic tracks, including TF ChIP-seq, from up to one megabase of DNA and provides variant scoring through a free API for non-commercial use. Evo 2 [42] is a DNA foundation language model trained across all domains of life that scores variants by sequence likelihood and provides open weights and a hosted inference API. GPN-Star [43] is a phylogeny-informed genomic language model that leverages whole-genome alignments and species trees for genome-wide variant effect prediction and is released as open-source weights and code.

In the independent SNP-SELEX benchmark of [44], FABIAN-variant 2022 ranked fourth of 14 prediction models on both the First Batch (median per-TF AUROC 0.906) and the Novel Batch (0.689).

FABIAN-variant 2026 is, to our knowledge, the only tool that combines PWMs, TFFMs, and deep learning-derived models for variant scoring. It is also the only tool offering web, API, and standalone access, mouse genome support, support for indels and single nucleotide variants, known TFBS filtering from five data sources, and heterodimer TF complex predictions.

## Materials and methods

### Model database

TF names from all sources are mapped to canonical gene symbols and assigned to human and/or mouse using annotations from HOCOMOCO [8] and CIS-BP [7]. Models matching both species are included in both organism sets. A heterodimer complex is included for an organism only if both of its component TFs are annotated for that species. All models undergo preprocessing (zero-column trimming and a 30 bp maximum length filter). Position count matrices and position frequency matrices are then converted to PWMs as described in [45] using the background nucleotide distribution of the respective species. Sequence logos for both PWMs and TFFMs are generated through a unified LaTeX/TikZ pipeline, so that they are directly comparable on the results page.

### PWM and TFFM scoring

For each selected PWM or TFFM, FABIAN-variant evaluates the motif at every position overlapping at least one variant base, on both strands, and retains the maximum score per sequence. This yields a reference score $s_\mathrm{ref}$ and a variant score $s_\mathrm{alt}$, each in $[0, 1]$. The per-model score $S$ is computed as in the previous version of FABIAN-variant:


\begin{eqnarray*}
S = \tanh(F \cdot \ln 2), \qquad F = \frac{s_\mathrm{alt} - s_\mathrm{ref}}{1 - \max(s_\mathrm{ref},\, s_\mathrm{alt}) + \alpha}
\end{eqnarray*}

with pseudocount $\alpha = 0.1$. $S$ ranges from $-1$ (predicted loss of binding) to $+1$ (predicted gain). The combined score per variant–TF pair is the average of per-model $S$ values, using only TFFM scores when available (configurable on the results page).

### BPNet deep learning scoring

The BPNet scorer is implemented in C++. Given a 2114 bp genomic sequence centred on the variant position, it computes for each TF and each cell-line model the predicted ChIP-seq binding profile and total read count, averaged across both strands, for the reference and variant sequences. From these predictions, it derives (i) the log_2_ fold change (logFC) in read counts, quantifying the overall change in binding strength, and (ii) the Jensen–Shannon distance (JSD) between the binding profiles, capturing changes in the spatial distribution of binding. Per-cell-line values are returned without aggregation (Fig. 2c).

### Combined score against SNP-SELEX

**Figure 3.**
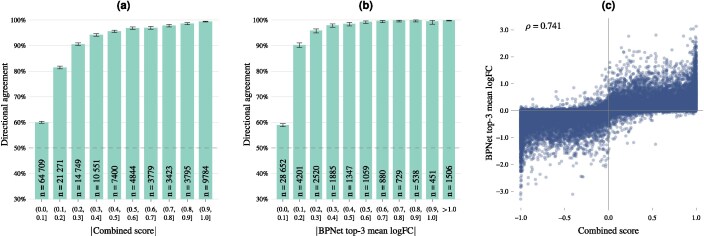
Agreement of FABIAN-variant 2026 predictions with SNP-SELEX [46]. (**a**) Directional agreement of the combined score with $-$PBS per |combined score| bin (PBS-significant, nonzero combined score; $n = 144\;305$). Bars show Wilson 95% confidence intervals. The dashed line marks the chance level (50%). (**b**) Same analysis for the BPNet score per |BPNet top-3 mean logFC| bin, plus an overflow bin for values $> $1.0 (PBS-significant, nonzero BPNet top-3 mean logFC; $n = 43\;768$). (**c**) Pairwise relationship between the combined score and the BPNet top-3 mean logFC across the 38 751 PBS-significant variant–TF pairs with nonzero values in both scores (116 TFs; pooled Spearman $\rho = 0.741$).

The SNP-SELEX dataset [46] comprises $151\;289$ unique variants (GRCh37/hg19 coordinates). SNP-SELEX TFs were mapped to the FABIAN-variant TF panel by gene symbol, with HOCOMOCO synonyms as fallback (493 of 503 mapped). Variant–TF pairs with a SNP-SELEX preferential binding score (PBS) significant at $P < 0.05$ were scored with FABIAN-variant using PWMs from JASPAR and HOCOMOCO, and TFFMs from JASPAR. Combined scores per variant–TF pair (see the “PWM and TFFM scoring” section) were joined with the PBS (<0: variant-allele preference; >0: reference-allele preference). Directional agreement (fraction of pairs with matching combined-score and $-$PBS signs) was computed for ten 0.1-wide |combined score| bins from $(0.0, 0.1]$ to $(0.9, 1.0]$, excluding pairs with a zero combined score ($n = 144\;305$; Fig. 3a). Wilson 95% confidence intervals were computed per bin.

### BPNet against SNP-SELEX

For the BPNet-involving analyses (Fig. 3b and c), logFC values were aggregated per variant–TF pair as the mean logFC of the three cell-line models with the largest |logFC| (BPNet top-3 mean logFC). Directional agreement of this score against $-$PBS was computed on variant–TF pairs covered by both BPNet and SNP-SELEX (PBS-significant, nonzero BPNet top-3 mean logFC; $n = 43\;768$), binned by |BPNet top-3 mean logFC| in ten 0.1-wide bins plus an overflow bin for values $> $1.0 (Fig. 3b).

### Combined score versus BPNet

Spearman rank correlation between the combined score and the BPNet top-3 mean logFC was computed on variant–TF pairs covered by PWMs/TFFMs, BPNet, and SNP-SELEX (PBS-significant, nonzero in both scores; $n = 38\;751$; 116 TFs; Fig. 3c).

### Runtime comparison

End-to-end response times of FABIAN-variant 2022 and 2026 were measured on three representative workloads (single variant, 100-variant batch, 10 000-variant VCF) in a Playwright-driven headless Chromium browser, three runs each. To match the 2022 model counts (3790 PWMs and 1224 TFFMs), the 2026 model set was restricted to PWMs from JASPAR, HOMER, and hPDI, and TFFMs from JASPAR (3830 PWMs and 1290 TFFMs). BPNet deep learning scoring was disabled in the 2026 version (Table 2).

## Case examples

To illustrate FABIAN-variant's output, we selected five variants from published studies where the affected TF and direction of effect have been experimentally established (Table 3). The variants are located in promoters of disease-relevant genes. For all five, the predicted direction (loss or gain of binding) agreed with the experimental findings. Coordinates are given in GRCh38.

**Table 3.** Example promoter variants with experimentally confirmed effects on TF binding

dbSNP ID	Gene	TF	Score	Effect	Ref.
rs2814778	*ACKR1*	GATA1	–0.9960	loss	[47]
rs968567	*FADS2*	ELK1	+0.9701	gain	[48]
rs2104934553	*PROC*	HNF1A	–0.9136	loss	[49]
rs587776528	*PIGM*	SP1	–0.5815	loss	[50]
rs3219018	*FCGR2B*	FOS::JUN	–0.2557	loss	[51]

The combined score ranges from −1 (loss of binding) to +1 (gain of binding). Each score was generated with FABIAN-variant default settings and links to the corresponding detailed results page.

The *ACKR1* promoter variant chr1:159204893 T$>$C (rs2814778) abolishes GATA1 binding and erythroid gene expression [47]. The *FADS2* promoter variant chr11:61828092 C$>$T (rs968567) facilitates ELK1 binding and increases promoter activity [48]. Both variants are shown on the results page (Fig. 1). FABIAN-variant predicted strong loss across the entire GATA family for the *ACKR1* variant and a gain across the ETS family for the *FADS2* variant.

The *PROC* promoter variant chr2:127418425 T$>$C (rs2104934553) disrupts a hepatocyte nuclear factor 1 binding site [49]. The HNF1A detailed results page is shown (Fig. 2). FABIAN-variant predicted strong loss for HNF1A (combined score $-0.9136$). The two BPNet models available for HNF1A, both trained on HepG2, also predicted strong loss.

The *PIGM* promoter variant chr1:160032009 G$>$C (rs587776528) disrupts SP1 binding [50]. FABIAN-variant predicted a moderate loss; five of six SP1 TFFMs predicted loss while one predicted a gain. The *FCGR2B* promoter variant chr1:161662856 G$>$C (rs3219018) decreases FOS::JUN binding [51]. FABIAN-variant predicted a moderate loss; the score reflects the absence of a TFFM for this heterodimer.

## Validation against SNP-SELEX

We compared FABIAN-variant’s predictions against the PBSs from SNP-SELEX [46]. For PBS-significant variant–TF pairs (SNP-SELEX $P < 0.05$), directional agreement between the combined score and $-$PBS rises with |combined score|: 60.0% at $(0.0, 0.1]$, 81.4% at $(0.1, 0.2]$, and 99.4% at $(0.9, 1.0]$ (Fig. 3a). The BPNet score shows the same pattern, rising from 58.9% for the weakest predictions to ~99.9% for the strongest (Fig. 3b). The combined score (motif-based) and the BPNet score (CNN on ChIP-seq profiles) are independently derived. A pairwise comparison of the two scores over the 38 751 PBS-significant variant–TF pairs with nonzero values in both scores (116 TFs) yields a strong pooled rank correlation (Spearman $\rho = 0.741$; Fig. 3c). Large-magnitude predictions in either method reliably indicate loss or gain, while small scores should be interpreted cautiously.

## Conclusion

FABIAN-variant 2026 is a substantial update to the original application [1]. The approximately eight-fold expansion of the prediction model library and the addition of mouse genome support broaden the range of TFs and species for which variant effects can be predicted. Deep learning predictions now complement PWM/TFFM-based scoring and can capture binding effects beyond the motif. The expanded known TFBS sources and heterodimer complex support provide additional biological context for variant interpretation. The rewritten backend, RESTful API, and standalone version improve performance and accessibility for both interactive and programmatic use.

## Outlook

The new Rust web server and modular per-datasource architecture make FABIAN-variant easier to extend with new database releases than the 2022 version.

PWM and TFFM scoring currently uses a single CPU core per job, and we are considering multicore scoring to accelerate large VCF jobs. BPNet inference already uses multiple CPU cores. Adding GPU acceleration would further reduce runtime and enable the integration of larger deep learning models that capture longer sequence context or cell-type-specific chromatin features.

## Data Availability

FABIAN-variant 2026 is available at https://fabianapp.org/variant26/. The web server is free and open to all users and there is no login requirement. Results are accessible via a unique URL and retained on the server for 14 days. Users can save a project to prevent automatic deletion, or delete their data immediately. A tutorial with example data is provided. Documentation for the API and standalone version is available on the web server. FABIAN-variant is part of the GeneCascade Software Suite (https://www.genecascade.org/).
